# Characterisation of the DNA sequence specificity, cellular toxicity and cross-linking properties of novel bispyridine-based dinuclear platinum complexes

**DOI:** 10.1186/s12885-016-2368-0

**Published:** 2016-05-25

**Authors:** Ben W. Johnson, Vincent Murray, Mark D. Temple

**Affiliations:** School of Science and Health, Western Sydney University, Campbelltown, NSW 2560 Australia; School of Biotechnology and Biomolecular Sciences, University of New South Wales, Sydney, NSW 2052 Australia

**Keywords:** Anticancer drug, Cisplatin, DNA adducts, Interstrand cross-linking, Sequence specificity, Linear Amplification Reaction, Telomeric repeat

## Abstract

**Background:**

The anti-tumour activity of cisplatin is thought to be a result of its capacity to form DNA adducts which prevent cellular processes such as DNA replication and transcription. These DNA adducts can effectively induce cancer cell death, however, there are a range of clinical side effects and drug resistance issues associated with its use. In this study, the biological properties of three novel dinuclear platinum-based compounds (that contain alkane bridging linkers of eight, ten and twelve carbon atoms in length) were characterised to assess their potential as anticancer agents.

**Methods:**

The properties of these compounds were determined using a DNA template containing seven tandem telomeric repeat sequences. A linear amplification reaction was used in combination with capillary electrophoresis to quantify the sequence specificity of DNA adducts formed by these compounds at base pair resolution. The DNA cross-linking ability of these compounds was assessed using denaturing agarose gel electrophoresis and cytotoxicity was determined in HeLa cells using a colorimetric cell viability assay.

**Results:**

The dinuclear compounds were found to preferentially form DNA adducts at guanine bases and they exhibited different damage intensity profiles at the telomeric repeat sequences compared to that of cisplatin. The dinuclear compounds were found to exhibit a low level of cytotoxicity relative to cisplatin and their cytotoxicity increased as the linker length increased. Conversely, the interstrand cross-linking efficiency of the dinuclear compounds increased as the linker length decreased and the compound with the shortest alkane linker was six-fold more effective than cisplatin.

**Conclusions:**

Since the bifunctional compounds exhibit variation in sequence specificity of adduct formation and a greater ability to cross-link DNA relative to cisplatin they warrant further investigation towards the goal of developing new cancer chemotherapeutic agents.

## Background

Cancer is one of the most common public health threats of the 21^st^ century. Globally, it is the leading cause of death in economically developed countries and the second leading cause of death in developing countries [1], with about 14.1 million cancer cases and 8.2 million cancer-related deaths estimated to have occurred in 2012 [2]. Whilst this statistical overview of cancer is considerably bleak, there is a vast amount of promising research being undertaken, with a primary focus on the development of novel chemotherapy agents for use in the clinical treatment of cancer.

**Fig. 1:**
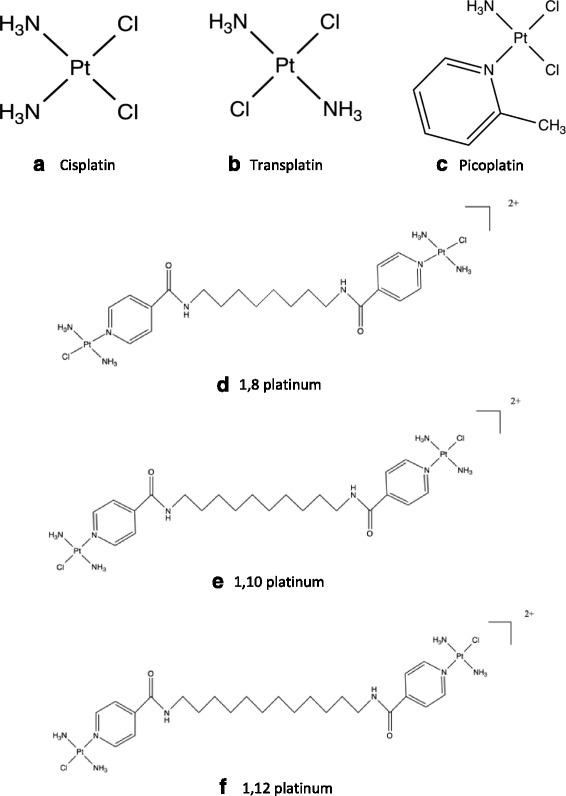
The chemical structures of cisplatin, transplatin, picoplatin and the three novel dinuclear platinum-based compounds used in this study. Note that (**a**) cisplatin, (**b**) transplatin and (**c**) picoplatin are mononuclear compounds, consisting of a single central platinum atom; whereas the (**d**) 1,8 platinum, (**e**) 1,10 platinum and (**f**) 1,12 platinum are dinuclear compounds, consisting of two platinum atoms separated by alkane linker chains containing 8, 10 and 12 carbon atoms, respectively. Note that picoplatin is shown for reference only and was not used in this study

*cis-*diamminedichloro-platinum(II) (cisplatin) is an FDA-approved, platinum-based drug that has been identified as a significant breakthrough for use in cancer chemotherapy, particularly in the treatment of testicular, cervical, ovarian, head, neck and, small and non-small cell lung cancer [3]. It is a square planar, mononuclear compound consisting of a central platinum atom, as shown in Fig. 1 (a). Unfortunately, there are a range of associated cytotoxic side effects associated with cisplatin’s clinical use, such as neurotoxicity, nephrotoxicity and emetogenesis [4, 5]. In an attempt to resolve these unwanted consequences, a range of novel platinum-based analogues have been made and tested. There is a general consensus that DNA is a key biological target of cisplatin [6–9], whereby the platinum drug binds predominantly to the N7 position of purine bases (guanine and adenine) [10], resulting in the formation of monofunctional and bifunctional DNA adducts. The majority of DNA adducts occur as a result of cisplatin binding to adjacent purine bases on the same strand of the DNA helix, as intrastrand DNA adducts. It is thought that DNA intrastrand adducts at GG sequences are the significant sites of cisplatin’s biological effectiveness and anti-tumour activity [11, 12]. The 1,2 GG intrastrand adduct is the most common type of DNA adduct formed by cisplatin and accounts for approximately 60 % of all DNA adducts; followed by the 1,2 AG intrastrand adduct, 1,3 GG intrastrand adduct and the GG interstrand adduct, which account for 20, 5 and 2 % of all DNA adducts, respectively [10].

In eukaryotic cells, telomeres consist of G-rich sequences, consisting of tandem repeats of 5′-TTAGGG-3’ [13, 14] and in immortalised cells, such as cancer cells, the telomeres do not shorten in a normal manner after cell division has occurred, due to the activity of the telomerase enzyme maintaining the length of the telomeres [15, 16]. Since cisplatin preferentially forms DNA adducts at consecutive guanines, the telomeres have been shown to be effective targets for cisplatin binding [17–19]. Furthermore, the cisplatin-induced DNA adducts have been shown to effectively cause telomere loss in HeLa cells [20] and in patients with advanced head and neck cancer; ultimately resulting in tumour regression [21] Hence, the ability of a drug to form DNA adducts at consecutive guanine bases of telomere sequences, is a crucial property to consider in the development of novel platinum-based anticancer drugs.

Platinum anti-cancer drugs remain one of the most widely used family of agents in the treatment of human cancer. Despite their success, clinical efficacy is often impeded by severe dose-limiting side effects [22–24]. The limited doses that can be administered to patients also means that tumors can develop resistance [25]. New drugs continue to be developed to overcome this issue.

In this investigation we have focused primarily on the DNA sequence specificity and DNA cross-linking ability of novel platinum complexes, in addition to investigating their cytotoxicity in HeLa cells. DNA interactions were determined using an automated DNA sequencer which is more precise for measuring the location and intensity of platinum-DNA adduct formation compared to prior slab gel approaches. As such, three novel dinuclear platinum-based complexes, *trans-*Diamminedichloroplatinum(II)-*N,N’*-octane-1,8-diyl)bis(isonicotinamide) (1,8 platinum), *trans*-Diamminedichloroplatinum(II)-*N,N’*-(decane-1,10-diyl)bis(isonicotinamide) (1,10 platinum) and *trans*-Diamminedichloroplatinum(II)-*N,N’*-(dodecane-1,12-diyl)bis(isonicotinamide) (1,12 platinum), have recently been synthesised [26]. The 1,8 platinum, 1,10 platinum and 1,12 platinum compounds contain alkane bridging linkers (separating the two platinum moieties) of eight, ten and twelve carbon atoms in length, respectively, as shown by their chemical structures in Fig. 1 (d) to (f). The platinum groups on either end of the alkane linker are derived from cisplatin’s related *trans*-configured isomer, *trans*-Diamminedichloroplatinum(II) (transplatin), shown in Fig. 1 (b). The novel compounds were designed as dinuclear trans-oriented platinum complexes derived from isonicotinic acid and transplatin joined by a variable length linker, to build upon the DNA binding profile of this distinct class of anticancer agents. It is thought that these will bind in a different way to the mononuclear cisplatin, transplatin and structurally similar picoplatin (without the 2-methyl group), as shown in Fig. 1. As a case in point, the trinuclear trans-oriented platinum complex, BBR3464 is up to 1000-fold more cytotoxic in vitro than cisplatin and is able to overcome acquired resistance in a panel of human cancer cell lines [27, 28] as discussed further in [26]. It should also be pointed out that picoplatin contains a methyl group, which protects it from attack by biological nucleophiles, such as cysteine and methionine containing peptides/proteins [29–31], and attains a higher nuclear concentration compared with both BBR3464 and cisplatin [32], however, isonicotinic acid was not methylated in this present study.

An assessment of the biological properties of novel platinum-based drugs is a crucial first step to predict their potential for use in the clinical treatment of cancer. In the long term, it is anticipated that a knowledge of platinum-based anti-tumour properties will enable the design and synthesis of a range of platinum-based drugs with desirable chemotherapeutic properties that surpass cisplatin’s clinical efficacy.

This article reports the biological properties of the three novel dinuclear platinum-based compounds, through the application of various molecular biology-based techniques. More specifically, this investigation characterises the sequence specificity of DNA adducts induced by the novel dinuclear platinum-based compounds, using a purified G-rich DNA template containing seven sites of telomeric repeat sequences. Additionally, the interstrand cross-linking efficiency (ICLE) of the dinuclear compounds, using a purified DNA template, and their cytotoxicity in human tissue culture cells, have been characterised.

There are a range of methods that can be utilised to determine the sequence specificity of cisplatin and related analogues, such as the linear amplification reaction (LAR) (combined with automated DNA sequencing) [33] and single-strand ligation PCR (sslig-PCR) [34]. The LAR in particular, is advantageous for detecting bulky DNA adducts, which involves *Taq* DNA polymerase extending from a radioactively- or fluorescently-labelled oligonucleotide primer until its activity is terminated by a DNA adduct [35–37]. Thermal cycling allows the adduct-containing DNA templates to be linearly amplified, forming truncated DNA fragments that are dependent on the sites of DNA adduct formation. The same labelled primer and untreated DNA template are used to carry out dideoxy sequencing reactions, which provide size standards that enable the precise determination of compound sequence specificity. Previously, the LAR was routinely carried out with a radioactive ^32^P-labelled oligonucleotide primer and the compound sequence specificity was analysed from a DNA sequencing gel [38, 39]. However, more recently cisplatin’s DNA sequence specificity has effectively been assessed by the LAR procedure, through the incorporation of a fluorescently-labelled primer, which is subsequently analysed by capillary electrophoresis with laser-induced fluorescence (CE-LIF) [40]. Furthermore, the use of CE-LIF, in place of the conventional gel electrophoresis, is a faster method and the data can be quantified more accurately [41, 42].

Interstrand cross-links formed by cisplatin are less abundant than the intrastrand adducts, occurring at a frequency of approximately 6 % [43]. They are the most toxic type of DNA adduct, that induce strong local distortions in the double helix and inhibit the separation of the DNA strands within cells [44]. This hinders normal DNA metabolic processes such as DNA replication and transcription and subsequently causes cell cycle arrest and apoptosis [45, 46]. These DNA damaging effects associated with cisplatin-induced interstrand cross-links, are a desirable property in the continued development of novel platinum-based antitumour drugs. A compound’s ICLE can effectively be determined through the application of a denaturing agarose gel assay. In particular, an alkaline-based denaturing agarose gel has commonly been employed to assess the interstrand cross-linking ability of various platinum-based compounds [47, 48]. The denaturing agarose gel carried out in this current investigation was adapted from a novel urea-based denaturing agarose gel assay [49]. This particular assay has the ability to effectively separate long double-stranded DNA (dsDNA) templates (ranging from 1 to 23 kb in length) into their single-stranded DNA (ssDNA) constituents. The dsDNA and ssDNA conformations can easily be distinguished on the denaturing agarose gel, as the ssDNA band exhibits a lower apparent molecular weight than the dsDNA band. This characteristic is particularly advantageous for characterising the ICLE of cisplatin and other novel covalent-binding compounds, as any compound-induced interstrand cross-links will prevent the DNA from denaturing. This effect can be observed on the denaturing agarose gel as a visible retention of the dsDNA. Through the application of this assay to this current investigation, the ICLE of the dinuclear platinum-based compounds were assessed, using a 2364 bp linearised pUC19 plasmid template.

Lastly this investigation reports the cytotoxicity of the novel dinuclear platinum-based compounds, which were assessed in the cervical cancer cell line, HeLa. Cervical cancer is the second largest cause of cancer-related mortality in woman and was responsible for more than 200,000 deaths in 2010, with 46,000 of these deaths occurring in the 15 to 49 age group [50]. It has been shown that the combination of bevacizumab (a monoclonal antibody to vascular endothelial growth factor) with cisplatin and radiotherapy, is generally a safer form of cervical cancer treatment, with only 31 % of patients experiencing adverse side effects [51]. However, improvements can still be made to improve the clinical effectiveness of platinum-based drugs towards cervical cancer with reduced adverse side effects. It is for this reason that there is a need for the continued characterisation of novel platinum-based compound cytotoxicity and their associated intracellular effects within cervical cancer cells. The tetrazolium (MTT) and sulforhodamine B colourimetric assays are commonly utilised to assess the cytotoxicity of novel platinum-based compounds. More recently, the MTT assay was employed to determine the cytotoxicity of a novel bifunctional dinuclear platinum-based compound in a human ovarian carcinoma cell line, whereby it was determined that the two monofunctional ends of the dinuclear compound were converted to bifunctional cross-links at a slower rate than cisplatin [52], contributing to its reduced cytotoxicity. The MTT assay was similarly employed in this study, in order to determine the cytotoxicity (IC_50_) of the novel dinuclear compounds in HeLa cells. Whilst the sulforhodamine B assay has been reported to be more sensitive than the MTT assay, both assays have been shown to produce comparable results to determine IC_50_ values [53]. The broad goal of such in vitro and cell based experimental approaches is to be able to screen new compounds quickly and efficiently as a pipeline to assess their potential use in further clinical trials and to assess their possible risks. One of the problems of drug development is the relatively slow transition of new cisplatin analogues to the clinic and hence the need for further studies such as this, that aim to apply modern molecular approaches to assess the potential of novel analogues for therapeutic use in humans.

## Methods

### Chemicals and starting materials

Cisplatin and transplatin were purchased from Sigma-Aldrich. The novel dinuclear platinum-based compounds, 1,8 platinum, 1,10 platinum and 1,12 platinum, were designed by Nial Wheate of the University of Sydney, Australia and synthesised as described in [26]. Cisplatin and transplatin were dissolved in dimethylformamide (DMF) to give working stock solutions of 5 mM. The dinuclear compounds were dissolved in DMF to give a working stock solution of 1 mM, as these compounds were poorly soluble at higher concentrations. For the cytotoxicity experiments, the dinuclear compounds were dissolved at a concentration of 1 mM in DNase-free water to resolve complications associated with DMF-induced cytotoxicity in cells treated at high drug concentrations.

DH5α *E. coli* cells transfected with pUC19 containing an insert of seven telomeric repeat sequences (pUC19/T7), inserted between the *Bam*HI and *Hin*dIII restriction enzyme sites [40]. This clone pUC19/T7 plasmid was used for all DNA-drug interactions in this study.

The 5′ FAM-labelled reverse sequencing primer (FAM-REV) [40] used in the linear amplification procedure, was purchased HPLC purified from Invitrogen at a 50 nmole scale, consisting of a 5′–3′ sequence of AACAGCTATGACCATG (16 bases long).

The denaturing agarose buffer used in the interstrand cross-linking procedure, consisted of 0.5 mg/mL bromophenol blue, 8 M urea, 1 % (v/v) tergitol nonyl phenoxypolyethoxylethanol type-40 and 1 mM tris(hydroxymethyl)aminomethane (Tris) (pH 8) in DNase-free water. An Amresco 1 kb DNA ladder (without loading dye) was prepared by adding 9 μL of denaturing loading buffer to 0.5 μL of the 1 kb ladder, followed by 0.5 μL of DNase-free water. The denaturing agarose gel was prepared as a 1.2 % (w/v) agarose gel containing 1 M urea. The 1 X TAE denaturing gel running buffer consisted of 40 mM Tris-acetate, 1 mM ethylenediaminetetraacetic acid (EDTA) and 1 M urea (pH 8).

### DNA preparation

The pUC19/T7 clone DNA was extracted and purified from the DH5α *E. coli* cells, using a Qiagen Plasmid Maxi purification kit. The purified plasmid pUC19/T7 clone was linearised with a *Pvu*II restriction enzyme prior to the DNA damage experiments. Following the restriction digest, the DNA was concentrated via ethanol precipitation and the resulting DNA pellet was resuspended in an appropriate volume of 10 mM Tris-HCl (pH 8.8), 0.1 mM Na_2_EDTA to give a DNA stock concentration of no less than 100 ng/μL.

### DNA damage reactions

DNA damage reactions were carried out by treating 800 ng of the *Pvu*II-cleaved pUC19/T7 DNA with various concentrations of each compound. The samples were prepared in a final reaction volume of 40 μL, consisting of 2 mM *N*-2-hydroxyethylpiperazine-*N’*-2-ethane sulfonic acid (HEPES) (pH 7.8), 10 mM NaCl and 10 μM EDTA, incubated at 37 °C for 18 h in the dark. A DMF solvent control was prepared and incubated under the same conditions as the drug-treated samples by substituting the drug with 5 % (v/v) DMF. This control was used to assess any negligible background damage due to potential solvent effects caused by the DMF-dissolved compounds. After incubation, an ethanol precipitation was carried out on all samples and the resulting DNA pellets were re-dissolved in 20 μL of 10 mM Tris-HCl (pH 8.8), 0.1 mM Na_2_EDTA.

### Linear amplification reaction and dideoxy sequencing

The sites of DNA damage were determined by subjecting the drug-treated, *Pvu*II-cleaved pUC19/T7 plasmid samples to the LAR. In a 20 μL final reaction volume, 48 ng of the drug-treated plasmid DNA was mixed with 1 pmol of the FAM-REV primer, 67 mM Tris-HCl (pH 8.8), 16.6 mM (NH_4_)_2_SO_4_, 6.7 mM MgCl_2_, 0.3 mM dNTPs and 1 U of *Taq* DNA polymerase [18]. This was then made up to volume with DNase-free water. The samples were subjected to 20 cycles of 95 °C for 45 s, 57 °C for 1 min, 72 °C for 45 s, and a final extension of 72 °C for 5 min in a Bio-Rad DNA Engine Dyad Peltier Thermal Cycler. The reaction products were then ethanol precipitated for 1 h on ice to remove artifact DNA fragments, followed by centrifugation at 20,000 x g for 30 min. The DNA pellets were washed twice with 150 μL of 70 % (v/v) ethanol prior to drying the DNA pellets and re-suspending in 10 μL of 10 mM Tris-HCl (pH 8.8), 0.1 mM Na_2_EDTA. The reaction products were analysed by submitting a 2 μL aliquot to the Ramaciotti Centre, University of New South Wales, Sydney, Australia, where it was analysed on an Applied Biosystems ABI 3730 Capillary Sequencer. Dideoxy sequencing reactions were carried out on the same DNA template using the same reaction components as the LAR, but with the addition of 1 mM of ddNTPs (ddCTP was used in this study to produce its complementary G sequence trace) and 50 μM of dNTPs. The dideoxy samples were subjected to the same reaction conditions and ethanol precipitation procedure as the LAR samples, and submitted for analysis as described for the LAR samples above. All LAR experimental results were consistently reproduced at least three times for each of the tested compounds.

### Interstrand cross-linking assay

A volume of 25 μL of denaturing agarose gel loading buffer was combined with 5 μL of drug-treated, *Pvu*II-cleaved pUC19/T7 DNA. Two DMF negative controls were prepared in the same manner, but with the substitution of the drug-treated DNA with 5 % (v/v) DMF-treated DNA. The drug-treated samples were heat denatured at 95 °C for 10 min, along with one of the DMF controls (heat denatured ssDNA control). The 1 kb ladder and remaining DMF control (non-heat denatured dsDNA control) were placed on ice. The 1.2 % (w/v) denaturing agarose gel was cast with a Bio-Rad Wide Mini-Sub Cell GT System and submerged in 1 X TAE/1 M urea buffer (pH 8). The entire volumes of each of the heat denatured samples and non-heat denatured sample, were loaded onto the denaturing agarose gel, along with 10 μL of the 1 kb ladder. Gel electrophoresis was carried out at 4.2 V/cm for 6 h, at 4 °C in the dark. This allowed the short 340 bp *Pvu*II*-*cleaved dsDNA fragments to run off the gel, whilst producing well resolved bands for the remaining 2364 bp dsDNA fragments (the largest linearised portion of the pUC19 plasmid after complete digestion with *Pvu*II). Following electrophoresis, the gel was washed at least three times with 1 X TAE buffer (pH 8), followed by staining in 1 X GelRed stain (diluted in 1 X TAE buffer (pH 8)) for at least 30 min [49]. The gel was visualised under UV light with a BioRad Gel Doc 2000 imager and analysed for compound induced cross-linking, using BioRad Quantity One gel imaging software to quantify the intensity of bands on the gel. The interstrand cross-linking assay was reproduced at least three times for each of the tested compounds.

### Preparation of HeLa cells for cytotoxicity assay

HeLa cells were cultured as sub-confluent monolayers in 75 cm^2^ culture flasks and maintained at 37 °C in 5 % CO_2_ in a Heal Force SMART CELL incubator. The cells were subcultured twice on a weekly basis in Dulbecco’s modified eagle medium (DMEM), supplemented with 10 % (v/v) Fetal bovine serum (FBS), 4.5 g/L D-Glucose, L-Glutamine, 110 mg/L Sodium Pyruvate, 200 U/mL Penicillin and 200 μg/mL Streptomycin. The HeLa cells were harvested for cell counting after allowing the cells to incubate at 37 °C in 5 % CO_2_ for 3 days, or until the cells had reached 90 % confluence. The DMEM media was decanted from the flask and the cells were trypsinised upon the addition of 5 mL of 0.25 % (w/v) Trypsin-EDTA. Following trypsinisation, 5 mL of fresh DMEM was added to the flask and this final 10 mL solution was collected in a Falcon tube, which was then centrifuged at 500 x g for 5 min to pellet the cells. The media was decanted and the cell pellet was washed with 10 mL of Dulbecco’s phosphate buffered saline (DPBS), followed by centrifugation at 500 x g for 5 min. The DPBS was decanted and the cell pellet was resuspended in 10 mL of fresh DMEM media. A 10 μL aliquot of the DMEM cell suspension was combined with 10 μL of 0.4 % (w/v) trypan blue solution and the trypan blue/cell suspension was mixed thoroughly prior to loading a 10 μL aliquot onto a BOECO haemocytometer for cell counting. The number of cells per mL was calculated and the 10 mL cell suspension was diluted with DMEM media to yield a final concentration of 100,000 cells/mL. This diluted cell suspension was then plated in 100 μL aliquots into the wells of a flat-bottomed 96-well microtitre plate so that each well of the 96-well plate contained 10,000 cells. The 96-well plate was placed into the incubator and maintained at 37 °C with 5 % CO_2_, for 24 h.

### Cytotoxicity assay

The DMEM culture media in the wells of the 96-well plate were removed by pipette and replaced with 100 μL of fresh DMEM media containing cisplatin or novel drug analogue at concentrations of 1, 3, 5, 10, 30, 50 and 100 μM. For the novel dinuclear compounds, the drug concentration was extended further to 150, 200 and 300 μM. All drug treatments were carried out in triplicate and untreated cell controls (cells in DMEM without drug) were included for each experiment. DMF solvent controls (cells in DMEM treated with no higher than 2 % (v/v) DMF, which corresponds to the amount of DMF present in cells treated with 100 μM cisplatin) were also included in triplicate for each experiment. Triplicate water controls, consisting of 70 % DMEM and 30 % DNase-free water, were included for the dinuclear compounds, to account for any loss in cell viability induced by the water present at drug treatments of 300 μM. Empty wells (containing no cells) were filled with 100 μL DMEM media, in triplicate, for the DMEM media blank controls. The 96-well plate was then placed back into the incubator and maintained at 37 °C with 5 % CO_2_, for 24 h.

Following drug treatments, the cells were treated with MTT by adding 50 μL of the MTT-DPBS solution to the cells in each well of the 96-well plate. Following a 2 h incubation at 37 °C with 5 % CO_2_, the media was removed from the wells by pipette and the resulting formazan crystals were solubilised in 100 μL of DMSO [54]. The 96-well plate was placed on an IKA MTS 2/4 digital microtitre plate shaker for 30 min to ensure thorough solubilisation of the formazan crystals prior to measuring the optical density at 550 nm with a Thermo Scientific Multiskan EX microplate reader.

## Results

### Sequence specificity of the dinuclear platinum-based compounds in the pUC19/T7 sequence

**Fig. 2:**
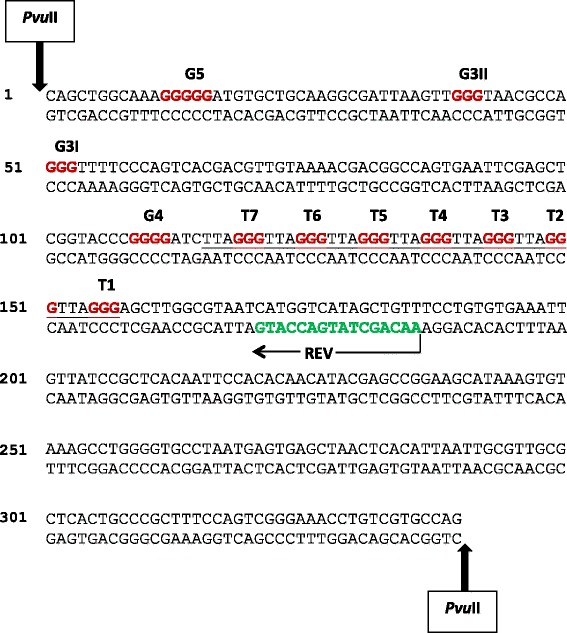
Double-stranded sequence of the *Pvu*II-cleaved pUC19/T7 DNA template. The upper strand of the sequence is written in the 5' to 3' direction. Sites of *Pvu*II restriction enzyme cleavage are indicated by the bold vertical arrows at both ends of the sequence and the annealing site of the REV primer is shown by a horizontal arrow, with the primer sequence highlighted in green. The seven telomeric repeats sequences (T1 to T7) are underlined, with the guanines highlighted in red. All other sites of three or more consecutive guanines (G4, G3I, G3II and G5) are highlighted in red

A modified pUC19/T7 plasmid was utilised for the DNA sequence specificity assays carried out in this investigation. The pUC19/T7 sequence was composed of seven telomeric repeats consisting of TTAGGG (T1-T7), a site of four consecutive guanine bases (G4), two sites of three consecutive guanine bases (G3I and G3II) and a site of five consecutive guanine bases (G5), as highlighted in Fig. 2. After treating separate *Pvu*II-cleaved pUC19/T7 DNA samples with each compound, the LAR procedure was carried out with the FAM-REV, which typically yields a 186 bp full length ssDNA product. The annealing site of the FAM-REV is indicated within the *Pvu*II-cleaved pUC19/T7 sequence in Fig. 2. The ssDNA products generated by the LAR procedure were analysed on an ABI 3730 capillary sequencer by fragment analysis, alongside reaction products obtained from dideoxy sequencing on the untreated *Pvu*II-cleaved pUC19/T7 DNA.

**Fig. 3:**
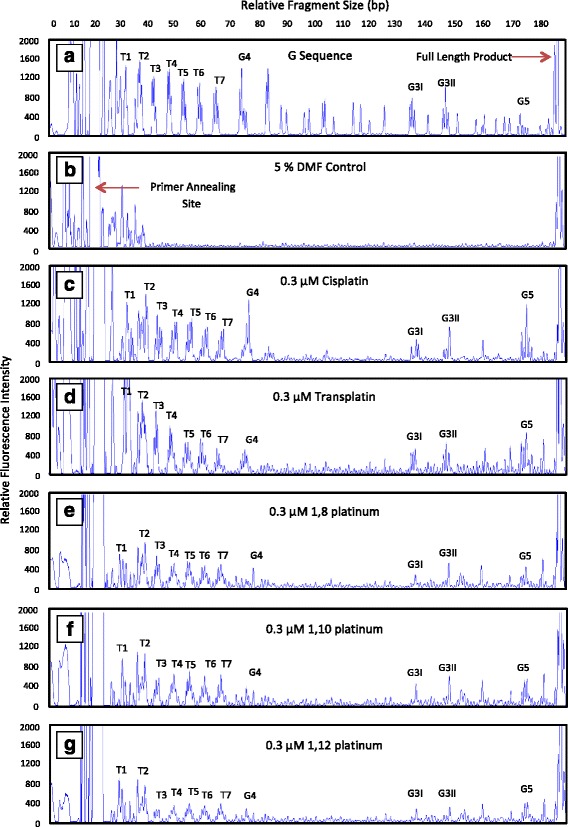
Images of LAR electropherograms showing DNA damage induced by cisplatin, transplatin and the novel dinuclear compounds in the *Pvu*II-cleaved pUC19/T7 sequence. The top two electropherograms represent the (**a**) G sequence (generated via dideoxy sequencing and used as a reference for determining the location of all G-rich sites in the sequence) and the (**b**) 5 % DMF control (without drug). The following five electropherograms show a trace of peaks corresponding to DNA damage induced by (**c**) 0.3 μM cisplatin, (**d**) 0.3 μM transplatin, (**e**) 0.3 μM 1,8 platinum, (**f**) 0.3 μM 1,10 platinum and (**g**) 0.3 μM 1,12 platinum. For each electropherogram image, the relative fluorescence intensity of the peaks is plotted on the y axis and the relative DNA fragment size (bp) plotted on the x axis (shown at the top of the G sequence electropherogram). The peak corresponding to the full length extension product occurs at the expected size of 186 bp in all electropherograms, which is highlighted in the G sequence electropherogram. The primer annealing site occurs at the left of each electropherogram, as indicated in the 5 % DMF control. The sites of the seven telomeric repeats (T1 to T7) are indicated, as well as the other G-rich sites (G4, G3I, G3II and G5) at which DNA adducts occur

**Fig. 4:**
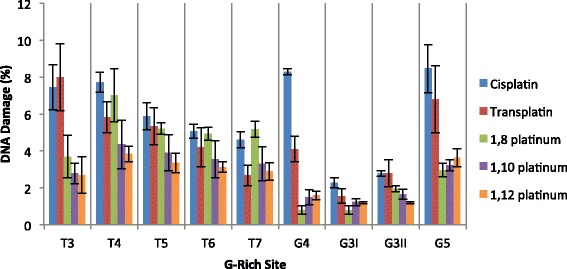
Graph highlighting the percentage of DNA damage induced by each compound at the major G-rich sites in the *Pvu*II-cleaved pUC19/T7 sequence. The percentage of overall DNA damage is plotted on the y axis and the nine major G-rich sites plotted on the x axis. The percentage of DNA damage was determined for each compound at sites in the sequence containing at least three or more consecutive guanine bases. The total percentage of DNA damage at each G-rich site was calculated from the sum of the percentage of DNA damage at each individual base within the G-rich site. The error bars represent the SEM, determined from three separate experiments. Note that the T1 and T2 sites have been omitted as the DNA damage at these sites was unreadable due to artifact peaks present in the DMF control

The LAR experimental conditions were optimised by treating the DNA template with varying concentrations of novel dinuclear compound. An optimal compound concentration of 0.3 μM resulted in both the relatively even distribution of damage across the entire length of the DNA template, as shown in Fig. 3 (c) to (g), and the full length extension product of 186 bp (this corresponds to the end-point of the ssDNA fragment whereby extension by *Taq* DNA polymerase is terminated), as indicated in the G sequence electropherogram in Fig. 3 (a). DNA damage induced by the compounds can be observed predominantly at the telomeric repeat sequences, as well as at the other four G-rich sites in the *Pvu*II-cleaved pUC19/T7 sequence, as shown in Fig. 3 (c) to (g). For all tested compounds, any solvent effects caused by the DMF were found to be negligible compared to DNA damage caused by the compounds, as evidenced by the absence of background peaks in the DMF trace shown in Fig. 3 (b). Compound-induced DNA damage could not be interpreted at the first two telomeric repeat sites (T1 and T2) as a result of artifact peaks consistently present in the DMF controls, which appear to be attributed to their close proximity to the primer annealing site [40], as indicated in Fig. 3 (b). The telomeric repeat sites yielded the highest total percentage of DNA damage (collective sum of damage induced at T3-T7), attributing to 16–30 % of total DNA damage within the analysed pUC19/T7 sequence, thus indicating that they are effective targets for DNA adduct formation by both the mononuclear and dinuclear platinum compounds. The telomeric repeat sites, T5-T7, yielded the most similar amounts of DNA damage for each compound, with the level of DNA damage ranging between 3–6 %, as evident in Fig. 4. Relatively less DNA damage was observed at the G3I and G3II sites for all compounds, with damage at these sites not exceeding 3 %; whereas the G5 site attracted a higher level of damage, ranging from 3–8 %, as shown in Fig. 4. Interestingly, the 1,8 and 1,10 platinum compounds induced a level of DNA damage at the G5 site comparable to that induced at the T5-T7 sites, accounting for over 3 % of DNA damage, as evident in Fig. 4. However, cisplatin and transplatin induced DNA damage levels of 7–8 % at the G5 site; approximately two-fold higher than damage induced by the dinuclear compounds. Furthermore, it can be noted that cisplatin and transplatin induced the highest levels of DNA damage at the G4 site, accounting for approximately 8 and 4 % of DNA damage, respectively. In contrast, the dinuclear compounds induced low levels of DNA damage at this same site, accounting for less than 2 % of total DNA damage.

**Fig. 5:**
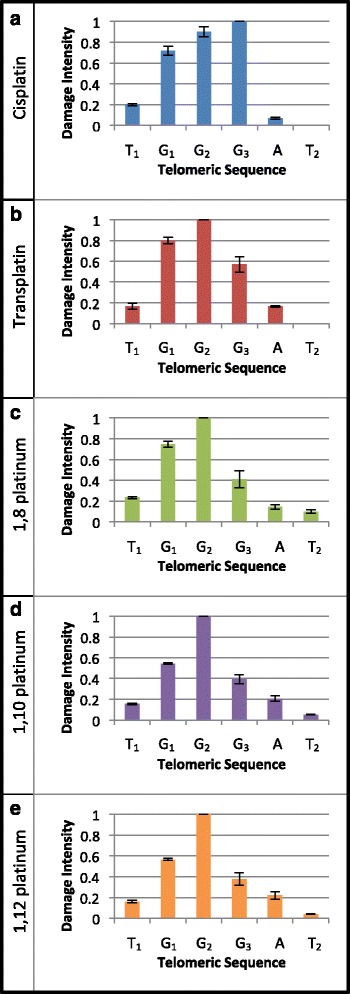
Graphs illustrating the damage intensity profile induced by each compound at the telomeric repeat sites in the *Pvu*II-cleaved pUC19/T7 sequence. The graphs for (**a**) cisplatin, (**b**) transplatin, (**c**) 1,8 platinum, (**d**) 1,10 platinum and (**e**) 1,12 platinum, are highlighted in blue, red, green, purple and orange, respectively. The damage intensity (normalised to a value of up to 1, whereby a value of 1 was assigned to the base that yielded the most intense damage) was determined from the percentage of damage at each base in the telomeric sequence. The error bars represent the SEM, determined from three separate telomeric repeat sites – T4, T5 and T6. The six nucleotides of the telomeric repeat are referred to as T_1_G_1_G_2_G_3_AT_2_

A subsequent analysis was conducted to further characterise each compounds sequence specificity; paying particular attention to their damage intensity profiles at the telomeric repeat sites in the pUC19/T7 sequence. For each of these sites, the percentage of damage at each base was determined using GeneMapper software and normalised to a maximal value of 1, relative to the highest percentage of damage. The damage intensities were averaged from three telomeric repeat sites, T4, T5 and T6, as these particular telomeric repeats produced the most consistent damage intensity trends across three repeat experiments for each compound tested. Cisplatin was found to induce the largest intensity of apparent damage at the third G (G_3_) in the sequence, followed by (in decreasing order) the second guanine (G_2_), first guanine (G_1_), the first thymine (T_1_) and adenine (A), as shown in Fig. 5 (a). It can be noted that no apparent damage was induced by cisplatin at the second thymine (T_2_) in the sequence. Transplatin displayed a different damage intensity profile to that of cisplatin, whereby the most intense apparent damage was induced at G_2_, followed by (in decreasing order) G_1_, G_3_, A and T_1_, as shown in Fig. 5 (b). Interestingly, all three dinuclear compounds yielded similar damage intensity profiles at the telomeric repeat sites. The 1,10 platinum and 1,12 platinum compounds produced almost identical damage intensity profiles, with the highest intensity of apparent damage being induced at G_2_, followed by (in decreasing order) G_1_, G_3_, A, T_1_ and T_2_, as evident in Fig. 5 (d) and (e). The 1,8 platinum compound produced a relatively similar damage intensity profile to that of 1,10 platinum and 1,12 platinum, but with a notably higher proportion of apparent damage being induced at G_1_, as evident in Fig. 5 (c). In summary, it can be noted that the unique damage intensity profiles determined for the novel dinuclear compounds, do not match the profile obtained for cisplatin; rather their damage intensity profile is a close match to that of transplatin, with the exception being that apparent damage was detected at the T_2_ base for the dinuclear compounds (transplatin did not induce apparent damage at this base).

### Interstrand cross-linking efficiency of the dinuclear platinum-based compounds

The extent of drug-induced DNA cross-linking was determined by measuring the change in ratio of ssDNA to dsDNA, in each lane of the denaturing agarose gel. These changes were observed across a drug concentration gradient ranging from 0.01 to 30 μM. The concentration at which the compounds induced a 50 % retention of the dsDNA form (i.e. the drug concentration that prevents 50 % of the dsDNA from being denatured to the ssDNA form on the denaturing agarose gel), was determined through non-linear regression analysis, as carried out with GraphPad Prism software. This measure is a useful indicator of how effectively a novel drug can cause DNA cross-linking and was analysed with respect to the reference compound, cisplatin. The frequency of interstrand cross-linking was calculated according to the formula, %ICL/Pt = XL/5408 x r_b_ [55], whereby %ICL/Pt refers to the percentage of interstrand cross-links per platinum adduct, XL (XL = -ln *A*, where A is the fraction of DNA molecules running as a band corresponding to the non-cross-linked DNA) is the number of interstrand cross-links per molecule of the linearised DNA template and 5408 is the number of nucleotide residues. The r_b_ ratios were estimated from the molarity of platinum compound and nucleotides present in the solution at which 50 % of the DNA was resistant to denaturing. This assumes that all of the drug has reacted with the DNA. This assumption is supported by the fact that whilst determining the sequence specificity, at a 10-fold higher drug concentration, there were still undamaged DNA sequences present since adding more platinum compound led to further damage that interfered with the polymerase extension reaction. The ICLE value for each compound is displayed in Table 1, whereby the higher the compounds ICLE value, the greater its DNA cross-linking efficiency.

**Table 1:** Summary of the interstrand cross-linking efficiency of the dinuclear platinum compounds experimental values and is reported along with its corresponding SEM value

Compound	ICLE (%)	Drug concentration at 50 % dsDNA retention (μM)	r_b_ (drug : nucleotide) at 50 % dsDNA retention	Last detected data point (μM)
Cisplatin	6.8 ± 0.5	0.09 ± 0.01	1.9E-3 ± 1.4E-4	10.0
1,8 platinum	43.5 ± 19.0	0.02 ± 0.01	4.7E-4 ± 3.1E-4	0.3
1,10 platinum	23.1 ± 3.9	0.03 ± 0.01	5.7E-4 ± 9.6E-5	0.3
1,12 platinum	14.2 ± 6.4	0.06 ± 0.02	1.1E-3 ± 5.0E-4	1.0

The compounds have been arranged vertically in the table alongside their corresponding ICLE values, expressed as a percentage. The drug concentration (expressed in μM) that induced a 50 % retention of dsDNA, is reported alongside its respective r_b_ value. The amount of nucleotide sample incubated with each compound was 2 x 10^-9^ moles, whereby this value was used to calculate the r_b_ value. The ‘last detected data point’ refers to the highest drug concentration whereby the DNA was still detectable on the agarose gel. All values are reported in the table with their respective SEM values, which were determined from three separate experiments

**Fig. 6:**
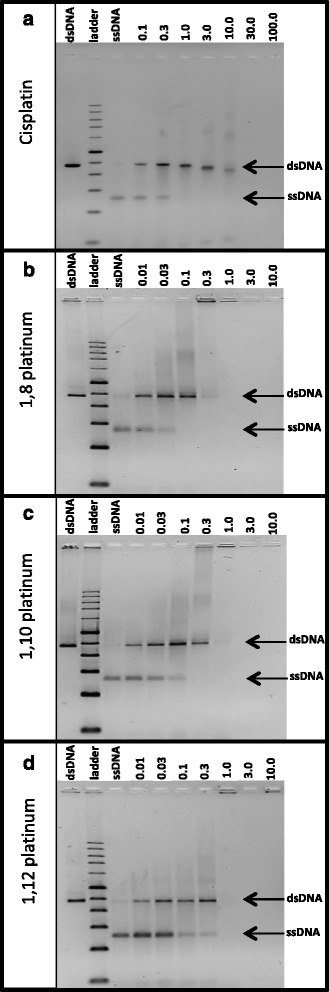
Images of 1.2 % denaturing agarose gels showing compound-induced cross-linking of double-stranded, *Pvu*II-cleaved pUC19 DNA. The denaturing agarose gel images depict bands corresponding to the large (2364 bp long) *Pvu*II*-*cleaved pUC19 DNA fragment following treatment with (**a**) cisplatin, (**b**) 1,8 platinum, (**c**) 1,10 platinum and (**d**) 1,12 platinum. The bands corresponding to the non-heat denatured dsDNA and heat denatured ssDNA forms (of the DMF control samples) are present on the left and right-hand side of the 1 kb ladder, respectively, of each gel. In each gel image, the bands to the right of the ssDNA control, correspond to heat-denatured DNA treated with increasing concentration of compound, ranging from 0.1 to 100 μM for cisplatin, and 0.01 to 10 μM for the dinuclear compounds (as indicated above each gel image)

Two DNA samples treated with only 5 % DMF solvent were included as negative controls for all experiments, since DMF was present at this concentration in all compound-treated DNA samples. For one of these control samples, the DNA template was denatured into ssDNA by heating at 95 °C, in keeping with the procedural treatment of the drug-treated samples. The other control DMF sample was left non-heat denatured to indicate the running position of the dsDNA on the denaturing gel. The different running positions of the dsDNA and ssDNA forms of the 5 % DMF samples, are evident in the first and third lanes, respectively, of each denaturing agarose gel image; these are highlighted as ssDNA and dsDNA in Fig. 6 (a). The extent of cross-linking of the linear dsDNA template induced by cisplatin, 1,8 platinum, 1,10 platinum and 1,12 platinum, is also shown in the lanes to the right of the ssDNA control in Fig. 6 (a) to (d), respectively. The results obtained for cisplatin in this investigation, indicate that cisplatin-treated dsDNA was unable to denature into its ssDNA form at a drug concentration of 1 μM, as indicated by the complete absence of the ssDNA band in the denaturing agarose gel image in Fig. 6 (a). Furthermore, the denaturing agarose gels revealed that 50 % of the dsDNA form was retained at a cisplatin concentration of 0.09 ± 0.01 μM, yielding an ICLE of 6.8 ± 0.5 %, as indicated in Table 1.

An analyses of the results for the three dinuclear compounds indicate that the most extensive DNA cross-linking was exhibited by the 1,8 and 1,10 platinum compounds. These two compounds were found to cause the complete reduction of the ssDNA form at a lower drug concentration than was observed for cisplatin, at a drug treatment of 0.3 μM, as highlighted in the gel images in Fig. 6 (b) and (c). The 1,8 and 1,10 platinum compounds were found to induce a 50 % retention of the dsDNA form at drug concentrations of 0.02 ± 0.01 μM and 0.03 ± 0.01 μM, respectively, as shown in Table 1. Furthermore, 1,8 platinum yielded an ICLE of 43.5 ± 19.0 % and 1,10 platinum, an ICLE of 23.1 ± 3.9 %. It can be noted that the ICLE values determined for 1,8 and 1,10 platinum, are approximately 6-fold and 3-fold higher than cisplatin’s ICLE, respectively. The 1,12 platinum compound induced DNA cross-linking to a slightly lesser extent than the 1,8 and 1,10 platinum compounds, causing 50 % of the dsDNA form to be retained at a drug concentration of 0.06 ± 0.02 μM and yielding an ICLE of 14.2 ± 6.4 %, as indicated in Table 1. Out of the three dinuclear compounds, it can be noted that 1,12 platinum’s interstrand cross-linking profile is closest to that of cisplatin’s.

It can be noted that the novel dinuclear compounds caused a complete loss in band intensity, of both the ssDNA and dsDNA forms, at a low drug concentration of 1 μM; which was not observed for cisplatin. This appears to be due to extensive inter-duplex DNA cross-linking induced by the compounds, whereby these higher molecular weight DNA structures remained trapped in the top of the wells of the denaturing agarose gel. This is evidenced by the band intensities detected in the top of the gel wells at drug concentrations of 0.3 μM and 1 μM, as seen in the denaturing agarose gel images in Fig. 6 (b) to (d).

### Cytotoxic activity of the dinuclear platinum-based compounds in HeLa cells

The degree of cytotoxicity is based on their IC_50_ values, as determined from analyses of their dose response curves. The compound IC_50_ values were interpolated from the dose response curves using GraphPad Prism software. This involved a non-linear regression analysis in order to identify the drug concentration that induced a 50 % inhibition of cell viability. The lead compound, cisplatin, was utilised as a reference for the comparative evaluation of novel compound cytotoxicity, with an IC_50_ value of 9.3 ± 0.2 μM being obtained in this investigation, as indicated in Table 2. In contrast, the novel dinuclear compounds exhibited a relatively weak cytotoxic activity, with IC_50_ values of > 40 μM, > 80 μM and > 130 μM for 1,12 platinum, 1,10 platinum and 1,8 platinum, respectively. Interestingly, it can be noted that the cytotoxic activity of the dinuclear compounds increase (correlating to a decrease in their IC_50_ value) with increasing length of their associated alkane linker, with the IC_50_ values approximately doubling with an increase in the alkane linker by two carbons. For instance, the 1,8 platinum compound contains the shortest linker of the three compounds and produced the highest IC_50_ value; almost double the IC_50_ of 1,10 platinum and almost four times higher than the IC_50_ value of 1,12 platinum.

**Table 2:** Summary of the dinuclear platinum compound-induced cytotoxicity in HeLa cells

Compound	IC_50_ (μM)
Cisplatin	9.3 ± 0.2
1,8 platinum	>130
1,10 platinum	>80
1,12 platinum	>40

The compounds are ordered vertically in the table alongside their corresponding IC_50_ values. These IC_50_ values represent the drug concentration, in μM, at which a cell viability of 50 % was obtained, and were determined from a non-linear regression analysis using GraphPad Prism software. Each IC_50_ value was determined from the average of three separate experiments

## Discussion

### Sequence specificity of the dinuclear platinum-based compounds in the pUC19/T7 sequence

We examined the DNA sequence specificity of three novel platinum-based dinuclear complexes, consisting of alkane linker chains of variable lengths, in a DNA sequence containing seven tandem telomeric repeats of TTAGGG and other clusters of G-rich sites, including a run of five consecutive guanines, GGGGG. Cisplatin was utilised as a positive control in this investigation, as it is known to target telomeric repeats and other guanine-rich sequences [18, 19, 40]. Transplatin was similarly used as a positive control for this study, as the novel dinuclear compounds contain *trans*-configured platinum groups at either end of their alkane linker, hence the sequence specificity of the dinuclear compounds could be effectively compared and contrasted to that of transplatin. The LAR was carried out, using a fluorescently tagged primer and CE-LIF, in order to determine the sites of compound-induced DNA damage (as a result of DNA adducts formed by the compounds). These data were quantified at base pair resolution using GeneMapper software.

As expected, the reference compound, cisplatin, was found to preferentially damage the pUC19/T7 sequence at guanine bases, particularly at the G-rich telomeric repeats (T1 to T7), G4, G5 and with less intense damage at the G3I and G3II sites. These results are in agreement with previous cisplatin investigations, which have similarly highlighted that human telomeric repeat sequences (as well as other G-rich sequences) are strong binding targets for cisplatin [18, 19, 40, 56]. The sites consisting of a larger cluster of guanine bases, such as the G5 site, attracted a higher level of DNA damage than sites with fewer clusters of guanine bases, such as the G3I site, which further supports cisplatin’s preference for G-rich sequences. Furthermore, the high damage intensities detected at the large clusters of consecutive guanine bases suggest the presence of 1,2 intrastrand GG DNA adducts, which is cisplatin’s most prevalent form of DNA adduct [5, 10, 57]. Additionally, cisplatin was found to cause DNA damage at adenine bases, particularly at the adenine (A) base following the third guanine base (G3) in the telomeric repeat sites, which suggests the presence of 1,2 GA intrastrand DNA adducts; cisplatin’s second-most common form of DNA adduct [5, 10, 57]. Interestingly, cisplatin induced a higher level of apparent DNA damage at the first thymine (T_1_) base of the telomeric repeats, rather than at the adenine (A) base, which is likely attributed to the *Taq* DNA polymerase pausing at this site as a result of extensive DNA damage attributed to DNA adduct formation at the following cluster of guanine bases (G_1_, G_2_ and G_3_) downstream.

Transplatin was found to exhibit an altered sequence specificity profile to that of its related isomer, cisplatin. This was particularly evident upon analysis of their differing damage intensity profiles at the telomeric repeats. This difference in sequence specificity between cisplatin and transplatin, is likely due to the fact that cisplatin forms bifunctional DNA adducts (through its two chloride leaving groups), whilst transplatin forms monofunctional DNA adducts (through one of its chloride leaving groups). These results are in agreement with a previous sequence specificity investigation, whereby differing DNA sequence specificity profiles were similarly determined for cisplatin and transplatin [58].

The novel dinuclear compounds, 1,8 platinum, 1,10 platinum and 1,12 platinum, were found to exhibit a unique DNA sequence specificity profile at the telomeric repeats of the pUC19/T7 sequence. This was evidenced by the fact that the dinuclear compounds induced a small amount of DNA damage at the second thymine (T_2_) base in the telomeric repeat site, which did not occur for cisplatin and transplatin. This could be attributed to the formation of a 1,3 intrastrand adduct between the third guanine (G_3_) and second thymine (T_2_) of the telomeric site or alternatively, this could be due to the formation of a 1,2 intrastrand adduct between the adenine (A) and the second thymine (T_2_) base. The latter postulation is in agreement with a recent sequence specificity study, whereby a range of dinuclear (similar in structure to the dinuclear compounds in this current study) and polynuclear platinum-based compounds were found to exhibit a strong binding affinity for adjacent adenine and thymine bases [59]. Furthermore, a chemical reactivity assay has previously identified a hypersensitivity of thymine bases to *trans*-configured dinuclear platinum compounds in a short oligonucleotide sequence, 5′-TGGT-3′, whereby unstacking of the 3′-T was also detected [60]. It is believed that the dinuclear platinum compounds atypical sequence specificity at pyrimidine bases, such as thymine and cytosine, is due to the length and flexibility of their associated linker chains, which enables the two platinum moieties to form long range DNA adducts [61–63]. For instance, it is likely that one of the platinum moieties on one end of the dinuclear compound, binds to a guanine base first, followed by the binding of the other platinum moiety on the opposite end of the compound. Hence, the first platinum moiety acts as an anchor, which mediates the binding of the remaining platinum moiety to a base within a restricted range in the DNA sequence, depending on the flexibility of the linker chain. It therefore seems plausible that this mechanism accounts for the higher proportion of DNA damage induced by the dinuclear compounds at thymine bases, as determined in this current study.

### Interstrand cross-linking efficiency of the dinuclear platinum-based compounds

The ICLE determined for the reference compound, cisplatin, yielded a value of 6.8 ± 0.5 % in this study. This value is in agreement with those previously published, whereby cisplatin’s ICLE has been determined to be approximately 6 % [43, 52]. The dinuclear platinum-based compounds, 1,8 platinum, 1,10 platinum and 1,12 platinum, yielded considerably higher ICLE values than that of cisplatin, with ICLEs ranging as high as 14 to 44 %. For instance, the ICLE of 43.5 ± 19.0 % determined for 1,8 platinum in this study, is approximately 6-fold higher than cisplatin’s ICLE. High interstrand cross-linking efficiencies, attributed to DNA adducts formed by dinuclear platinum-based compounds containing carbon-based linkers, are not uncommon and have similarly been reported for novel *cis*-configured platinum(II) complexes containing semi-rigid aromatic linkers [64], whereby they were found to yield high ICLE values ranging from 20 to 40 %. It can be noted that the ICLE values determined for the dinuclear platinum-based compounds in this current study, are in the vicinity of the ICLEs reported for the aromatic linker-containing platinum(II) complexes (mentioned above). Furthermore, high interstrand cross-linking efficiencies have similarly been demonstrated for a range of *trans*-oriented dinuclear platinum-based complexes containing variable long-chain aliphatic linkers [65], whereby ICLE values as high as 56, 72 and 91 % were determined for dinuclear platinum-based compounds consisting of 7, 10 and 12 methylene residues, respectively, in their aliphatic linker chains. It has therefore been proposed that the ICLE of dinuclear platinum-based compounds, increases in proportion to an increase in length of their aliphatic linker [65]. Interestingly, the trend observed in this current study is not in agreement with the conclusions of this previous study; rather the interstrand cross-linking efficiencies of the dinuclear platinum-based compounds in this current investigation were found to be inversely proportional to an increase in length of the carbon-based linker. An explanation for these contrasting results is that the chemical composition of the linker chain may also play a role in either facilitating or hindering the binding efficiency of these dinuclear compounds. For example, the dinuclear compounds assessed in this current study are composed of a carbon-based alkane linker, whereas the *trans-*oriented dinuclear compounds, containing variable long-chain aliphatic linkers (mentioned above), were synthesised with additional methylene and diethylenetriamine groups in their linkers. In support of this theory, a weak ICLE has previously been determined for a dinuclear platinum-based compound composed of a benzotriazolate linker [56]. Even though this benzotriazolate-based compound was reported to consist of two platinum moieties at either end of the linker (similar to the dinuclear compounds analysed in this current study), it was characterised as having a relatively low ICLE value of approximately 6 %. This supports the concept that the bulky aromatic ring of azole, from which the reported linker was composed, hinders its flexibility and attributes to the compounds poor efficiency to form interstrand cross-links. This indicates that the length and rigidity of the linker must also be considered in the design of the dinuclear compounds. However, it is not possible to predict the activity of a compound based solely on its structure, since the relationship between structure and activity is still not fully understood. For instance, this has been demonstrated for mononuclear platinum-based compounds (similar to cisplatin), whereby the incorporation of unconventional chemical groups have been shown to improve their ICLE; producing high ICLE values above 20 %, upon the incorporation of isopropylamine or azide groups [47, 66].

In general, it was noted that there was an accumulation of DNA sample in the wells of the gel, evident at dinuclear compound concentrations of 0.3 μM and above. This is thought to be due to extensive DNA cross-linking between multiple DNA duplexes, producing high molecular weight DNA structures that are incapable of migrating out of the wells of the gel. Such a trend has similarly been reported for the novel dinuclear complex, BBR3535 [55]. In addition to this, a reduction in DNA band intensity of the ssDNA and dsDNA forms could also be attributed to a reduction in the intercalating ability of the GelRed nucleic acid stain due to a high frequency of DNA adducts, formed by the two platinum moieties on either end of the compound. This effect has previously been characterised for ethidium bromide, whereby its intercalating activity was found to decrease markedly for DNA pre-damaged with a range of dinuclear platinum-based compounds [65]. Overall, the high ICLE values indicates that these compounds are markedly more effective at inducing a higher frequency of interstrand DNA cross-links than mononuclear compounds such as cisplatin.

### Cytotoxic activity of the dinuclear platinum-based compounds in HeLa cells

The IC_50_ value determined for cisplatin in this investigation was found to be in the order of 9.3 ± 0.2 μM. This value is in the same vicinity as the IC_50_ value of 11.4 μM that was recently determined for cisplatin, which was similarly assessed in HeLa cells through the application of the MTT assay [67]. The dinuclear platinum-based compounds, 1,8 platinum, 1,10 platinum and 1,12 platinum, were found to be relatively less cytotoxic to the HeLa cells in comparison to cisplatin, as evidenced by their markedly higher IC_50_ values. The dinuclear platinum-based compounds used in this study exhibited limited solubility in DMF and water, additionally they tended to precipitate after a 5 day period. Consequently, fresh stock of these compounds were used for each experiment since their precipitation would reduce the compounds molarity in solution. Poor aqueous solubility and stability properties of novel platinum-based compounds, containing bulky ligand groups, are a common issue that can interfere with the compounds ability to exert their cytotoxic activity. For instance, a range of bulky novel oxaliplatin-derived compounds have been shown to be less soluble in water than cisplatin as a result of their long carbon chains [68]. Hence, this indicates that the poor solubility associated with the dinuclear platinum-based compounds used in this current study is also due to the long carbon chains. One promising study has focused on the synthesis of platinum-based compounds with properties that aim to circumvent this compound solubility issue, such as through the substitution of chloride anions with diverse carboxylate anions, which promotes the aqueous solubility of the compounds and therefore the compound is available to interact with DNA in solution [69].

An interesting trend that was noted for the dinuclear platinum-based compounds in this current study, was an increase in cytotoxic activity with increasing size of the alkane linker bridging the platinum atoms. As highlighted in the results, the 1,12 platinum compound (containing the longest alkane linker) was found to be the most cytotoxic of the three dinuclear compounds, followed by the 1,10 platinum compound (containing the second longest alkane linker) and the 1,8 platinum compound (containing the shortest alkane linker). This trend has similarly been reported by a study that analysed the cytotoxicity of novel dinuclear bifunctional platinum-based complexes containing aliphatic linker chains of varying lengths, within two human ovarian carcinoma cell lines, A2780 (cisplatin-sensitive) and A2780cisR (cisplatin-resistant) [65]. The results from this previous investigation indicated that the activity of these dinuclear compounds increased with growing length of the aliphatic linker in both cell lines, with the compound containing the longest linker, exhibiting a comparable cytotoxicity to that of cisplatin in both cell lines. Hence, the cytotoxicity trend reported by this previous study are consistent with the trend reported for the dinuclear platinum-based compounds in this current investigation; albeit the cytotoxicity of 1,12 platinum was not found to be equivalent to cisplatin in this study. These findings support the potential design and synthesis of dinuclear platinum-based compounds with longer alkane linkers, such as a 1,14 platinum compound, whereby it seems plausible to assume that such compounds would induce an equivalent or improved cytotoxicity in comparison to cisplatin, however, their reduced solubility in water would likely pose an issue. It may be possible to improve the water solubility of these long-chain linkers through the incorporation of hydrophilic groups such as carboxylate groups in the linkers or in the leaving groups of the platinum complex [69].

## Conclusions

The sequence specificity, ICLE and cytotoxic activity of three novel dinuclear platinum-based complexes, 1,8 platinum, 1,10 platinum and 1,12 platinum, were assessed through the application of the LAR procedure, denaturing agarose gel and MTT assay, respectively. The sequence specificity analysis revealed that the novel dinuclear compounds preferentially damage the DNA at G-rich sequences, making the telomeric repeats a promising candidate for sites of DNA adduct formation in cancer cells. Furthermore, these compounds were found to exhibit a unique sequence specificity profile, inducing DNA damage at an additional thymine base at the telomeric repeat sites, not detected for cisplatin or transplatin. This altered sequence specificity appears to be attributed to the flexible alkane linker, which is a key component of the dinuclear compounds chemical structure. These compounds show good potential as anti-cancer agents in regards to their efficient interstrand cross-linking of DNA, exhibiting an ICLE of up to 6-fold higher than cisplatin. As interstrand DNA adducts are the most cytotoxic form of DNA adduct, this is a promising anti-tumour property to have. Despite the dinuclear compounds being capable of forming a high frequency of toxic DNA adducts, they exhibited a weak cytotoxic activity in the HeLa cells in comparison to cisplatin. Promisingly, an increase in their cytotoxic activity was found to be proportional to an increase in length of their associated alkane linker, which suggests that the future design of dinuclear platinum-based compounds with longer alkane linkers (whilst maintaining good water solubility), may have the potential to improve their cytotoxicity. The variation in sequence specificity of these bifunctional analogues in addition to their greater ability to cross-link DNA relative to cisplatin, indicates a spectrum of DNA lesions that may evade DNA repair and may form the basis of new cancer chemotherapeutic agents. However it remains to be seen if these compounds would be effective in a clinical situation and further cytotoxicity testing and cellular DNA binding studies, in various cancer cell lines, need to be carried out as part of the development and assessment of their clinical potential.

## Abbreviations

1,10 platinum, *trans*-Diamminedichloroplatinum(II)-*N,N’*-(decane-1,10-diyl)bis(isonicotinamide); 1,12 platinum, *trans*-Diamminedichloroplatinum(II)-*N,N’*-(dodecane-1,12-diyl)bis(isonicotinamide); 1,8 platinum, *trans-*Diamminedichloroplatinum(II)-*N,N’*-octane-1,8-diyl)bis(isonicotinamide); CE-LIF, Capillary electrophoresis with laser-induced fluorescence detection; Cisplatin, *cis*-Diamminedichloroplatinum(II); DMEM, Dulbecco’s modified eagle medium; DMF, Dimethylformamide; DPBS, Dulbecco’s phosphate buffered saline; dsDNA, Double-stranded DNA; EDTA, ethylenediaminetetraacetic acid; FAM-REV, 5′ FAM-labelled reverse sequencing primer; FBS, Fetal bovine serum; HEPES, *N*-2-hydroxyethylpiperazine-*N’*-2-ethane sulfonic acid; ICLE, interstrand cross-linking efficiency; LAR, linear amplification reaction; MTT, tetrazolium; ssDNA, single-stranded DNA; Transplatin, *trans*-Diamminedichloroplatinum(II); Tris, tris(hydroxymethyl)aminomethane
